# New Insights on In Vitro Maturation of Oocytes for Fertility Preservation

**DOI:** 10.3390/ijms251910605

**Published:** 2024-10-01

**Authors:** Flavie Gotschel, Charlotte Sonigo, Celeste Becquart, Ines Sellami, Anne Mayeur, Michael Grynberg

**Affiliations:** 1 Department of Reproductive Medicine and Fertility Preservation, Université Paris-Saclay, Assistance Publique-Hôpitaux de Paris, Antoine Beclère Hospital, 92140 Clamart, France; flavie.gotschel@aphp.fr (F.G.); celeste.becquart@aphp.fr (C.B.); ines.sellami@aphp.fr (I.S.); 2 Inserm, Physiologie et Physiopathologie Endocrinienne, Université Paris-Saclay, 94276 Le Kremlin-Bicêtre, France; 3 Histology-Embryology-Cytogenetic Laboratory, Université Paris-Saclay, Assistance Publique Hôpitaux de Paris, Antoine Beclère Hospital, 92140 Clamart, France; anne.mayeur@aphp.fr; 4 Department of Reproductive Medicine and Fertility Preservation, Hôpitaux Universitaires Paris-Seine-Saint-Denis, Assistance Publique-Hôpitaux de Paris, Jean Verdier Hospital, 93143 Bondy, France; 5 Unité Inserm U1133, Université Paris-Diderot, 75013 Paris, France

**Keywords:** in vitro maturation, fertility preservation, vitrification, oncofertility, cancer

## Abstract

In the last decade, the evolution of oncofertility has sparked a resurgence of interest in in vitro maturation (IVM) due to its suitability in certain oncological scenarios where controlled ovarian hyperstimulation may not be feasible. The retrieval of immature cumulus–oocyte complexes from small antral follicles, regardless of the menstrual cycle phase, presents a swift opportunity to vitrify mature oocytes or embryos post-IVM in urgent situations or when stimulation is not advisable. Harvesting immature cumulus–oocyte complexes and immature oocytes can be achieved transvaginally or directly in the laboratory from extracorporeal ovarian tissue. Although IVM has transitioned from an experimental status due to safety validations, it relies on the intricate process of oocyte maturation. Despite successful live births resulting from IVM in fertility preservation contexts, the comparatively lower developmental competence of in vitro matured oocytes highlights the necessity to enhance IVM culture systems. Recent advancements in IVM systems hold promise in bolstering oocyte competence post-IVM, thereby narrowing the gap between IVM and outcomes from ovarian stimulation. Additionally, for optimizing the chances of conception in cancer survivors, the combination of IVM and ovarian tissue cryopreservation stands as the favored choice when ovarian stimulation is unfeasible.

## 1. Introduction

In the history of infertility treatments, the in vitro maturation (IVM) of oocytes has been an important step. Indeed, IVM is a laboratory tool defined as the maturation in vitro of immature cumulus–oocyte complexes collected from small antral follicles. This concept of IVM was first described by Pincus and Enzmann [1] until the significant contribution of IVF pioneer Edwards [2], who observed that human oocyte GVBD occurred around 28 h and reached meiotic maturation after about 36 h [3]. From this point, Edwards focused on fertilizing the IVM human oocytes to produce human embryos, which was successfully achieved in 1969 [4,5]. With the race to achieve IVF, IVM has been abandoned. It took a while before the technique regained attention, as a consequence of the risk of ovarian hyperstimulation syndrome [6,7]. The lower developmental competence of oocytes matured in vitro when compared to the competence of their in vivo counterparts [8], combined with the emergence of innovative protocols of ovarian stimulation leading to an ovarian hyperstimulation syndrome free clinic, has induced another break in the development of IVM.

The two past decades have been marked by the groundbreaking emergence of female fertility preservation. Indeed, the major advances in cryopreservation techniques have allowed the possibility to freeze oocytes or ovarian tissue, opening a new era in the field of reproductive medicine. As a result, physicians had to take care of a new population, sometimes with severe diseases and urgent therapies to be started. In particular, some oncologic situations may not be compatible with 2 weeks of ovarian stimulation and/or the stimulation-induced hyperestradiolemia. In addition, ovarian tissue cryopreservation, often considered when stimulation cannot be considered, has its own limitations, especially concerns related to malignant cells reintroduction after graft. The development of oncofertility has provided a kind of “new life” for IVM, which filled the gaps of the two most conventional fertility preservation techniques. Hence, ASRM recently stated that IVM should no longer be considered experimental [9].

The present review aims to provide new insights in IVM for fertility preservation purpose.

## 2. Definition and Technical Aspects of IVM

The term “IVM” traditionally refers to the maturation process of immature cumulus–oocyte complexes (COCs) retrieved from small antral follicles and cultured from prophase I (germinal vesicle (GV) stage) through meiosis I to reach metaphase II (MII). These COCs are obtained from follicles not exposed to gonadotropins or preovulatory triggers, or minimal follicle stimulating hormone stimulation [2]. Like conventional IVF, there are a number of different variations on IVM, both in clinical practice and laboratory protocols [10]. Classically, standard IVM refers to the maturation in vitro of immature GV-stage intact COCs in one step to MII, from unstimulated or FSH-primed patients. Other protocols called hCG-triggered IVM consist of the maturation of COCs in vivo and in vitro in one step to MII, from patients triggered with hCG or a GnRH agonist.

Unlike matured oocytes obtained through hormonal stimulation, which exhibit a visible cumulus cell complex after ovulation, those from IVM present challenges in locating COCs due to their compact pre-ovulation form. COCs are identified in follicular fluid, rinsed, and incubated for 24 to 48 h in specialized in tissue culture like-medium supplemented with a protein source such as albumin or serum, follicle stimulating hormone (FSH), +/− other additives such as human chorionic gonadotropin (hCG), epidermal growth factor (EGF), estradiol, and/or cysteamine, under atmospheric oxygen for 1 to 2 days. The COC’s integrity as a cohesive functional entity is essential for the success of IVM [11,12,13].

After dissociation, cumulus–oocyte complexes undergo decoronization to remove cumulus cells via aspiration or pipette suction. Oocyte maturity is then assessed by polar body expulsion, followed by freezing using vitrification protocols or fertilization for embryo cryopreservation, similar to routine IVF.

The timing of oocyte pick-up has been reported after FSH priming, aiming to promote follicle growth. Ovarian stimulation with a few days of FSH priming is often used in clinical IVM programs. Animal studies have suggested that in vivo FSH priming enhances follicular development and the meiotic and developmental competence of immature oocytes and decreases the time required to reach the MII stage [14,15]. Likewise, in human IVM programs, FSH priming has been found to improve oocyte yield and maturation rates, resulting in more mature oocytes. The rationale for pretreating a patient with FSH is that human follicles with a diameter of 2–6 mm have a high expression of FSH receptors, and FSH augments follicular growth and estradiol production. Despite the rationale of FSH priming in IVM cycles, it is not clear whether a FSH priming alone, not combined with hCG, actually improves clinical outcomes in women without PCOS [16]. Some findings failed to find any improvement of FSH priming in IVM outcomes [17,18,19].

In addition, LH activity using hCG or GnRH agonist administration before immature oocyte collection has also been described to trigger oocyte maturation in vivo. However, the LH-induced dissociation of cumulus cells from the oocyte is, for some authors, an impediment in considering the further IVM process as actual IVM. Indeed, they claimed that IVM should not include the incubation of denuded oocytes, which is usually more related to rescue IVM, a procedure sometimes applied to immature oocytes collected after conventional ovarian stimulation and ovulation trigger. This practice is referred to as “Rescue IVM” and generally yields oocytes with poor developmental potential [20].

Several lines of evidence indicate that the diameter of human immature oocytes differs according to the patients’ age and the priming used before recovering the cumulus–oocyte complexes [21], relating to different mRNA storage and growth profiles in vitro [22]. Therefore, the source of immature oocytes may represent a crucial issue for oocyte IVM.

Standard IVM systems often yield lower rates of oocyte maturation in vitro compared to those retrieved through conventional IVF programs, where an ovulation trigger is administered. This suggests that many immature oocytes from small antral follicles might still lack the ability to mature meiotically and would have needed more time within their follicular environment for proper nuclear and cytoplasmic maturation. However, higher rates of oocyte maturation can be achieved by administering a bolus of hCG around 36–38 h prior to oocyte retrieval [23].

Despite ongoing debates about the most efficient clinical and laboratory protocols for patients undergoing IVM, there is no robust evidence favoring one system over the other (hCG-triggered or non-hCG-triggered).

The COC plays a crucial role in supporting the oocyte throughout its development. The oocyte stimulates granulosa cells to transform into cumulus cells to accomplish essential functions such as glycolysis to provide energy to the oocyte. The COC forms a three-dimensional complex with cumulus-to-oocyte projections that allow for the exchange of metabolites and regulatory molecules. This communication network, vital for oocyte development, is maintained until the post-LH period, when the oocyte enters the meiotic maturation phase. Preserving this communication is essential for successful IVF, as removing the COC from a follicle disrupts the cumulus-to-oocyte communication networks, compromising the developmental competence of the oocyte; oocytes are prone to spontaneous precocious maturation once removed from the follicle. This can be avoided by using meiotic inhibitors during oocyte retrieval. These inhibitors act on the COC and/or the oocyte to maintain the cumulus–oocyte communication network. These meiotic inhibitors are typically cAMP and/or cGMP modulators that can act on the cumulus cells and/or the oocyte to prevent activation of oocyte type 3A phosphodiesterase, thus preventing premature meiosis resumption. Collecting COCs for IVF in the presence of a meiotic inhibitor to preserve the delicate cumulus–oocyte communication network is an essential component of improving IVM [24].

Moreover, for successful fertilization and subsequent growth, it is crucial for the maturation of the oocyte cell’s nucleus and cytoplasm to be synchronized. Nuclear maturation involves GV breakdown induced by the LH surge followed by the resumption of meiosis and extrusion of the first polar body (MII). Cytoplasmic maturation refers to an accumulation of factors that prepare the cytoplasm for fertilization and embryonic development [3,25,26].

Therefore, recent discoveries regarding the inhibitors and regulators of oocyte maturation in animal models have contributed to improving outcomes in terms of live birth rates in MIV [27].

Natriuretic peptide precursor C (NPPC) in mural granulosa cells and it’s receptor NPR2 in cumulus cells play a role in maintaining high levels of cAMP in oocytes, which keeps them arrested in the meiosis cycle. LH triggers oocyte maturation by activating phosphodiesterase (PDE3A), which downregulates cAMP levels, leading to oocyte maturation. Estradiol promotes NPR2 expression, maintaining oocyte meiotic arrest [28].

**Figure 1:**
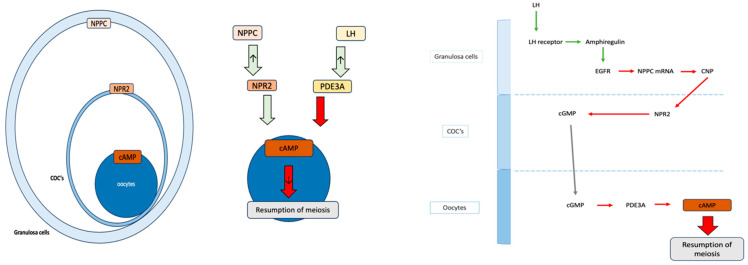
Regulation of oocyte meiotic arrest and resumption via NPPC, NPR2, cGMP, and cAMP pathways.

Evidence indicates that CNP acts as a key regulator of oocyte maturation, influencing the cell cycle and meiosis through its interaction with various cellular and molecular components, including NPR2 receptors and the levels of cGMP and cAMP (Figure 1). MicroRNAs like Let-7c influence gene expression in cumulus cells, affecting oocyte maturation. Various other factors, are also involved in oocyte maturation and division, necessitating further research for comprehensive understanding [29].

Recent advancements, particularly in a biphasic IVM system involving a prematuration step with meiotic arrest and cytoplasmic capacitation, followed by a second maturation step, have shown promising improvements in oocyte maturation rates and embryological outcomes, particularly for oocytes retrieved from small antral follicles [10].

## 3. Technique of Oocyte Retrieval

In IVM cycles, retrieving oocytes presents unique challenges compared to standard IVF collections. This is primarily due to smaller follicle sizes and the firmer attachment of immature cumulus–oocyte complexes to the follicle wall. Typically, the retrieval of immature oocytes is conducted transvaginally under ultrasound guidance, with the patient under general anesthesia, although local anesthesia is also an option. The needle diameter commonly used falls within the range of 16 to 21 gauge, while aspiration pressure typically varies from 80 to 120 mm Hg [30]. To prevent blood clot formation inside the aspiration needle, regular flushing with flushing media or the addition of 2 U/mL of heparin is usually performed. A retrospective cohort study comparing complication rates and pain scores between IVM and IVF cycles’ oocyte retrievals concluded that despite requiring more punctures per ovary and taking significantly longer than IVF collections, IVM retrievals did not exhibit a higher complication rate than standard IVF procedures [31].

Furthermore, immature cumulus–oocyte complexes can also be retrieved from ex vivo ovarian tissues or ovaries during a cesarean section. Indeed, ovarian tissue cryopreservation is another established method for female fertility preservation. It is particularly offered in pre-pubertal girls and also after puberty when time constraints do not allow for ovarian stimulation to harvest mature oocytes. Apart from avoiding hormonal administration, the ovarian tissue recovered for freezing contains many non-growing follicles, leading to possibilities of restoration of endocrine and exocrine ovarian function. More than 130 live births have been reported following ovarian cortex transplantation procedures, with a live birth rate per patient of approximately 25% [32,33].

This technique involves holding the tissues or ovaries with one hand while using a 5 to 20 mL syringe filled with buffered IVF media containing proteins, along with a 21- or 22-gauge needle for the aspiration process [34,35]. This method efficiently allows for the retrieval of small follicles containing immature oocytes.

## 4. From Polycystic Ovary Syndrome to Fertility Preservation: IVM in the Current Clinical Practice

IVM historically targeted patients with polycystic ovary syndrome (PCOS), providing a milder approach compared to controlled ovarian stimulation protocols [36]. Indeed, PCOS patients represent a population at increased risk of ovarian hyperstimulation syndrome, the most feared complication of ovarian stimulation. However, the development of multiple strategies to avoid the risk of ovarian hyperstimulation syndrome, i.e., GnRH agonist trigger in GnRH antagonist protocols and the freeze-all strategy, have hindered IVM interest and progress. The main reasons for pursuing IVM programs in PCOS women is the lower burden, reduced costs when compared to conventional IVF with ovarian stimulation, and patients who wish to limit hormonal treatments.

The role of IVM in female fertility preservation has been proposed more recently and well described in a recent review [37].

The primary source of data concerning IVM for fertility preservation in the medical literature stems from studies involving young women diagnosed with breast cancer. These investigations predominantly focus on oocyte pick-up (OPU)-IVM (Table 1), supplemented by research on ovarian tissue oocyte (OTO)-IVM (Table 2).

Embryo or oocyte cryopreservation stands as the most established technique for fertility preservation; however, ovarian stimulation to obtain in vivo mature oocytes might not suit all cancer patients. Indeed, the need to start cancer treatment urgently, or avoiding hyperestradiolemia, is by definition not compatible with conventional controlled ovarian stimulation protocols. In these situations, IVM emerges as a potential alternative, in particular for time issues, since the concern of high serum estradiol levels may now be counteract by anti-aromatase or tamoxifen administration during controlled ovarian stimulation [38].

The evidence indicates that small antral are permanently present in the ovaries of women of reproductive age, explaining that the yield of immature oocytes for further IVM may be considered regardless of the phase of the menstrual cycle [39]. Hence, Grynberg et al. reported a series of 248 breast cancer patients having undergone one IVM cycle for fertility preservation before chemotherapy, with a mean of 6.4 ± 0.3 mature oocytes cryopreserved [39]. The feasibility and safety of performing IVM in emergency settings has also recently been shown in women diagnosed with hematologic diseases [40].

Alternately to the usual transvaginal oocyte retrieval, immature cumulus–oocyte complexes can also be extracted from extracorporeal ovarian tissue [41]. This so-called ex vivo IVM has been associated with success [42,43] and may therefore be considered in theory as an additional source of gametes in all women undergoing oophorectomy or ovarian biopsies for ovarian tissue cryopreservation, except those who already received chemotherapy before ovarian tissue harvesting. The COCs are released upon dissecting the cortex from the medulla of the ovary, as small antral follicles are located in the junctional zone between the cortex and medulla. Although the number of COCs recovered from hemi- or whole ovary, this yield is much more limited when only small pieces of ovary are excised [44]. Another limitation of ex vivo IVM is related to the necessity of performing OTC in laboratories with specific expertise, which may require the transportation of the tissue if the surgical procedure is performed at a different location. Current OTC transportation protocols at 4 °C may be incompatible with ex vivo IVM requirements [45]. However, ex vivo IVM takes its advantage when the ovarian tissue is at high risk of malignant invasion, such as borderline ovarian carcinoma, leukemia, neuroblastoma and Burkitt lymphoma. Indeed, in these clinical situations OTC-IVM represents to date, the only safe method of fertility preservation, before the implementation of future techniques such as in vitro folliculogenesis [32,46]. Whatever the modality for obtaining immature oocytes, the evidence indicates that the vitrification should be performed after IVM. Indeed, despite similar survival rate between oocytes vitrified at GV or at M-II stage [47], the potential of oocyte maturation was significantly lower after the thawing of oocytes at the GV stage [48,49,50].

The IVM of oocytes continues to be developed as a pre-antral follicle culture and an in vitro follicular culture. Indeed, ovarian tissue cryopreservation is becoming increasingly important as a viable method for restoring fertility in girls and young women who are at high risk of sterility. However, concerns persist about the safety of transplanting cryopreserved ovarian tissue due to the potential risk of reintroducing cancer cells and triggering disease recurrence. To address these concerns, there is a need to develop culture systems that can support oocyte development from the primordial follicle stage. Despite the complexity of the folliculogenesis process, significant progress has been made in the in vitro growth of human follicles over the past decade. Currently, in vitro culture systems for ovarian tissue are based on two-dimensional substrates that fail to adequately support follicle survival or replicate the mechanical heterogeneity of the mammalian ovary. These systems, which include multi-well plates, microdrops, or membranes coated with extracellular proteins, have only been successful in producing live offspring from cultured primordial follicles in mice [51,52,53,54,55,56].

One of the primary challenges in follicular in vitro growth (IVG) is ensuring the growth of the primary follicle, along with the development of granulosa and theca cells, and the subsequent formation of an antrum. A critical limitation is the difficulty in maintaining the spherical structure of the follicle, which disrupts cellular interactions between the oocyte and granulosa cells, thereby compromising further in vitro development [57,58,59]. To address this, three-dimensional (3D) culture systems have been developed to resolve issues related to the spatial arrangement of cells. These systems, which enhance the diffusion of nutrients and gases through the follicles, have generated interest in advanced biomimetic models. The 3D culture techniques involve the use of homogeneous hanging drops or hydrogel encapsulation to maintain the follicular architecture. The culture of early antral follicles is strongly influenced by the composition and structure of the supporting tissue, highlighting the need for the development of extracellular matrices and biomaterials that mimic the ovarian physiological environment for optimal follicle development [60,61]. In addition to the spatial arrangement of cells, the extracellular matrix, a structural support network composed of collagen, laminin, and fibrinogen, is increasingly recognized as a key regulator in cell communication and differentiation [62]. Matrices that support follicle growth and maturation are essential for 3D culture systems. Various biomaterials, such as collagen, alginate, and Matrigel, have been explored as alternatives to mimic the ovarian extracellular matrix and to encapsulate and support human secondary and antral follicles. The stiffness gradient within the ovary clearly impacts follicular growth, but other physical properties of the matrix, such as composition and porosity, also need to be considered. The physical attributes of the 3D matrix used for IVG should be tailored to meet species-specific requirements. Thus, a precise culture system that facilitates the diffusion of nutrients and gases while retaining essential growth factors for oocyte maturation is needed. Currently, most incubations are based on static culture systems, with energy exchanges occurring every 24 to 72 h. However, tissue culture could benefit from dynamic systems, such as microfluidic or nanofluidic technologies, which allow for the configuration of a system that integrates specific steps of the process according to the needs of the cells. Additionally, within the ovary, there is increased vascularization deeper in the medulla, where secondary and antral follicles grow, indicating a greater need for nutrient diffusion during the final stages of maturation. Human ovarian cortex fragments cultured under flow conditions have shown improved follicle survival and growth [63]. However, these results are still preliminary, as the number of samples was low, and no matrix was used to maintain the 3D structure.

**Table 1:** Characteristics and results of studies reporting IVM of oocyte pick-up by transvaginal ultrasound–guided aspiration. (OPU-IVM).

Authors	Study Groups	No Patients	FP Indication	Age	AMH (ng/mL)	And AFC	Menstrual Cycle Phase	Priming	Oocytes Retrieved/Cycle	Oocytes Cryopreserved	Embryos Cryopreserved	Reutilization of Material
Sermondade et al. (2020) [64]	Total	17	BC	32.6 ± 5.0 ^c^	AMH 3.0 ± 1.7 ^c^	AFC 22.8 ± 8.7 ^c^	_	_	7.7 ± 4.5 ^c^	7.7 ± 4.5 ^c^	_	No
D’Hondt et al. (2020) [65]	Total	6	BC	_	_		_	_	_	16.6 ± 15.8 ^c^	2 ± 1.4 ^c^	Yes
Grynberg et al. (2020) [66]	Case report	1 (2 cycles)	AAP	36	AMH < 1	AFC 24	_	hCG	6 + 10	_	8	Yes
Rodrigues et al. (2020) [67]	Total	1	BC	32	_		FP	_	4	_	2	Yes
Sonigo et al. (2020) [40]	Total	25	HC	27.3 ± 6.1 ^c^	AMH 3.3 ± 3.2 ^c^	AFC 27.2 ± 14.1 ^c^	_	_	12 (6–15) ^d^	5 (4–10) ^d^	_	No
Delattre et al. (2020) [68]	Total	34	BC (n = 17), HC (n = 12), OC (n = 5)									
	OPU-IVM	17	BC (n = 8), HC (n = 7), OC (n = 2)	27.9 ± 6.6 ^c^	AMH 5.4 ± 7.6 ^c^	AFC 35.8 ± 29.4 ^c^	LP (n = 3) FP (n = 14)		_	9.2 ± 10.1 ^c^		Yes
	OPU-IVM+OTC+OTO-IVM	17	BC (n = 9), HC (n = 5), OC (n = 3)	25.9 ± 4.8 ^c^	AMH 5.7 ± 6.0 ^c^	AFC 34.3 ± 22.0 ^c^	_	_	_	3.9 ± 4.2 ^c (from OPU-IVM)^	_	Yes
Cohen et al. (2020) [69]	Total	158	BC (n = 108), HC (n = 23), OC (n = 27)	31 (27–35) ^d^	AFC 19 (13–25) ^d^			hCG	7.5 (4.8–13) ^d^	4 (2–8) ^d^	64 cycles for 104 patients	No
Sonigo et al. (2020) [70]	Total	172	BC								_	No
	Without injection	55	BC	31.5 ± 3.5 ^c^	AMH 3.0 ± 2.2 ^c^	AFC 20.5 ± 7.8 ^c^		-	9.1 ± 6.6 ^c^	5.1 ± 3.8 ^c^		No
	Priming by hCG	58	BC	33.3 ± 4.2 ^c^	AMH 3.2 ± 1.7 ^c^	AFC 20.5 ± 7.8^c^	_	hCG	9.2 ± 6.5 ^c^	5.4 ± 3.8 ^c^	_	No
	Priming by GnRHa	59	BC	31.2 ± 4.6 ^c^	AMH 2.8 ± 1.6 ^c^	AFC 19.9 ± 8.3 ^c^	_	GnRHa	9.1 ± 6.3 ^c^	6.0 ± 4.2 ^c^	_	No
Sermondade et al. (2019) [71]	Total	54	BC	30.8 ± 3.8 ^c^	AMH 1.9 (0.1–16) ^e^	AFC 12.5 (2–46) ^e^	_	_	4.0 (0–18) ^e^	3.0 (0–12) ^e^	_	No
Grynberg et al. (2019) [72]	Total	329	BC	32.1 ± 3.8 ^c^	AMH 4.0 ± 3.5 ^c^	AFC 21.5 ± 9.1 ^c^	_	_	_	_	_	No
	BRCA+ patients	52	BC	31.7 ± 3.9 ^c^	AMH 3.6 ± 2.9 ^c^	AFC 20.5 ± 11.4 ^c^	_	_	8.9 ± 6.9 ^c^	5.1 ± 3.8 ^c^	_	No
	BRCA-patients	277	BC	32.3 ± 3.8 ^c^	AMH 4.1 ± 3.6 ^c^	AFC 21.7 ± 12.1 ^c^	_	_	9.9 ± 8.1 ^c^	6.1 ± 5.1 ^c^	_	No
El Hachem et al. (2018) [73]	Total	373	BC (n = 329), HC (n = 15, OC (n = 29)	_	_		FP (n = 208) LP (n = 235)	GnRHa (n = 138) or hCG (n = 235)	_	_	Yes	No
	Priming by GnRHa	138	_	31.7 ± 4.4 ^c^	AMH 3.7 ± 2.4 ^c^	AFC 18.8 ± 7.6 ^c^	FP (n = 70) LP (n = 68)	GnRHa	9.1 ± 6.8 ^c^	5.2 ± 4.2 ^c^	Yes	
	Priming by hCG	235	_	32.2 ± 4.9 ^c^	AMH 3.6 ± 2.7 ^c^	AFC 18.6 ± 8.0 ^c^	FP (n = 138) LP (n = 97)	hCG	7.7 ± 5.5 ^c^	4.9 ± 4.0 ^c^	Yes	
Roesner et al. (2018) [74]	Case report	1	Anaplastic astrocytoma	24	AMH 7.89	AFC 40	_	_	13	8	_	No
Creux et al. (2018) [75]	Total	182	BC (n = 123), HC (n = 32), OC (n = 27)	30.6 ± 5.7 ^c^	AFC 17 (11.3–23) ^d^		FP (80.2%) LP (19.8%)	hCG	7 (5–12.5) ^d^	5 (2–8) ^d^	3 (2–5) ^d^	Yes
Creux et al. (2017) [76]	Total	164	BC (n = 113), HC (n = 26), OC (n = 25)	30 (27–35) ^d^	_			hCG	_	_	_	No
	Early Follicular Phase	46	_		17 (10–30) ^d^		FP	hCG	8.5 (4–15.8) ^d^	3 (0–7.3) ^d^	3 (2–5.8) ^d^	No
	Late Follicular Phase	107	_		17.5 (11.3–22) ^d^		FP	hCG	8 (5–14) ^d^	3 (0–7) ^d^	3 (0.5–5) ^d^	No
	Luteal Phase	39	_		17.5 (12.8–22.3) ^d^		LP	hCG	7 (4–9) ^d^	3 (1–5.5) ^d^	2 (1–3) ^d^	No
Hourvitz et al. (2015) [77]	Total	9	BC (n = 3), HC (n = 3), OC (n = 3)	27.2 ± 1.7 ^a^	_			FSH + hCG if time possible	12.3 ± 4.3 ^a^	7.3 ± 2.3 ^a^	2.0 ± 0.93 ^a^	No
Grynberg et al. (2016) [39]	Total	248	BC	31.5 ± 0.3 ^a^	AMH: 4.75 ± 0.33	AFC: 22.1 ± 0.8	FP (n = 127) LP (n = 121)	hCG	_	_	_	No
	Follicular Phase	127	BC	31.9 ± 0.4 ^a^	AMH 4.44 ± 0.4 ^a^	AFC 22.1 ± 0.8 ^a^	FP	hCG	9.3 ± 0.7 ^a^	6.2 ± 0.4 ^a^	7.8 ± 2.8 ^a^	No
	Luteal Phase	121	BC	31.0 ± 0.4 ^a^	AMH 5.03 ± 0.5 ^a^	AFC 22.9 ± 1.2 ^a^	LP	hCG	11.1 ± 0.8 ^a^	6.8 ± 0.5 ^a^	7.8 ± 2.0 ^a^	No
Sonigo et al. (2016) [78]	Total	340	BC (n = 300), HC (n = 14), OC (n = 26)	31.8 ± 4.5 ^c^	AMH 4.4 ± 3.8 ^c^	AFC 21.7 ± 13.3 ^c^	FP (49%) LP (51%)	hCG	9.5 ± 8.2 ^c^	5.5 ± 4.6 ^c^	Yes 39/340	No
Sonigo et al. (2016) [79]	Total	66	BC and HC	_	_		_	hCG	_	_	_	No
	Breast cancer	44	BC	29.5 ± 6.1 ^c^	AMH 2.7 ± 1.3 ^c^	AFC 17.0 ± 6.4 ^c^	_	hCG	5.5 ± 4.8 ^c^	3.5 ± 3.7 ^c^	_	No
	Hematological malignancies	22	HC	32.0 ± 4.3 c	AMH 2.9 ± 2.0 ^c^	AFC 18.0 ± 5.6 ^c^	_	hCG	8.5 ± 4.4 ^c^	6.0 ± 3.0 ^c^	_	No
Das et al. (2012) [80]	Total	9	BC (n = 5), HC (n = 3), OC (n = 1)	30.7 ± 2.1 ^a^	14 (9.5–17) ^d^		FP	hCG	6 (4–10) ^d^	_	_	No
Moria et al. (2011) [81]	Total	128	Cancer	_	_		_	_	_	_	_	No
	Breast cancer	87	BC	31.9 ± 0.5 ^c^	20(14–23) ^d^		_	_	9 (6–16) ^d^	_	_	No
	Hematological malignancies	16	HC	24.9 ± 1.1 ^c^	13(8–26) ^d^		_	_	8 (5–21) ^d^	_	_	No
	Gynecologic or abdominal malignancies	9	OC	29.5 ± 1.9 ^c^	16 (8–23) ^d^		_	_	6 (4–8) ^d^	_	_	No
	Other malignancies	16	OC	28.1 ± 1.3 ^c^	18 (16–22) ^d^		_	_	10 (5–15) ^d^	_	_	No
Maman et al. (2011) [82]	Total	18	BC (n = 5), HC (n = 5), OC (n = 5)	_	_		-	hCG	_	_	_	No
	Luteal Phase	5	_	23.4 ± 6.5 ^c^	AMH 3.7 ± 2.2 ^c^		LP	hCG	12.8 ± 8.4 ^c^	_	6.4 ± 6.6	No
	Follicular Phase	13	_	24.1 ± 5.4 ^c^	AMH 6.4 ± 6.9 ^c^		FP	hCG	17.3 ± 13.5 ^c^	_	7.8 ± 7.5	No
Huang et al. (2010) [83]	Total	38	BC	_	_		_	hCG	423	161	104	No
	Oocytes group	18	BC	33.1 ± 5.0 ^c^	_		_	hCG	237	161	_	No
	Embryo group	20	BC	34.7 ± 4.8 ^c^	_		_	hCG	186	_	104	No
Shalom-Paz et al. (2010) [84]	Total	66	BC	_	_		_	hCG	_	278	140	No
	Oocytes cryopreservation	35	BC	31.0 ± 5.2 ^c^	_		_	hCG	11.4 ± 8.8 ^c^	278	_	No
	Embryos cryopreservation	31	BC	34.2 ± 4.7 ^c^	_		_	hCG	9.7 ± 6.4 ^c^	_	140	No
Demirtas et al. (2008) [85]	Total	3	BC	21, 30, 40	_		_	hCG	53	36	2	No
Elizur et al. (2008) [86]	Total	5	Auto-immune disease	25.7 (19, 25, 26, 19, 35)	AFC 8, 23, 34, 14, 7		_	_	53	27	2	No

^a^: mean ± SEM, ^c^: mean ± SD, ^d^: median (IQR), ^e^: median (min-max). BC: breast cancer, HC: hematological cancer, OC: other cancer, OD: other disease, AAP: acute autoimmune polyendocrinopathy, LP: luteal phase, FP: follicular phase.

**Table 2:** Characteristics and results of studies reporting IVM of ovarian tissue derived oocytes (OTO-IVM).

Authors	Study Groups	FP Indication	N of Patients Undergoing OTO	Age	N of Patients with Retrieved Oocytes n(%)	N of Retrieved Oocytes	N of Retrieved Oocytes/Patient	Overall Maturation Rate		Oocytes Matured /Patient		Gametes Cryopreserved	Reutilization of Cryopreserved Material
Karavani et al. (2021) [87]	Pre-pubertal	HC (n = 7), OC (n = 26), OD (n = 7)	40	(1–5) ^e^ 4.2 ± 1.4 ^c^	13	117	9.0 ± 7.0 ^c^	4.3%	25.0%	0.4 ± 0.7 ^c^	2.6 ± 2.7 ^c^	O	No
				(6-menarche) 10.3 ± 1.6 ^c^	27	302	11.2 ± 6.2 ^c^	21.5%		2.4 ± 2.4 ^c^			
	Post-pubertal	HC (n = 39), OC (n = 47), OD (n = 7)	93	(menarche-17) 15.5 ± 1.4 ^c^	35	398	11.4 ± 8.5 ^c^	29.7%		3.4 ± 3.3 ^c^			
				(18–24) ^e^ 20.4 ± 2.0 ^c^	33	348	10.5 ± 7.4 ^c^	32.9%		3.6 ± 3.3 ^c^			
				(25–29) ^e^ 26.9 ± 1.2 ^c^	14	139	9.9 ± 11.7 ^c^	21.6%		2.1 ± 3.5 ^c^			
				(30–35) ^e^ 32.5 ± 1.8 ^c^	11	66	6.0 ± 6.4 ^c^	9.1%		0.5 ± 0.9 ^c^			
Delattre et al. (2020) [68]	Pre-pubertal	HC (n = 5), OC (n = 4)	9	5.1 ± 3.6 ^c^						3.7 ± 5.7 ^c^		O	Yes
	Post-pubertal OTC+OTO-IVM+COS	BC (n = 5), HC (n = 4), OC (n = 4)	13	26.2 ± 4.3 ^c^				41.4 ± 22.7%		6.7 ± 7.1 ^c^		O	
	Post-pubertal OTC + OTO-IVM	BC (n = 11), HC (n = 3), OC (n = 8), OD (n = 3)	25	27.9 ± 6.6 ^c^				34.8%		4.1 ± 3.7 ^c^		O	
Dietrich et al. (2020) [88]	Post-pubertal	BC (n = 6), HC (n = 3), OC (n = 3)	12	27.3 ± 4.8 ^c^	12/12 (100%)	36	3.1 ± 2.1 ^c^	38.9%		1.2 ± 0.7 ^c^		O	No
Cohen et al. (2020) [69]	Post-pubertal	Cancers	48	28.5 (21.5–34.4) ^d^	18	136	4 (3–11.8) ^d^	55.9%		3 (1.3–5.5) ^d^		O	No
Nikiforov et al. (2020) [45]	Post-pubertal	BC (n = 12), HC (n = 3), OC (n = 8), OD (n = 2)	25	28 (17–37) ^b^	25	895	36 (7–90) ^b^	31%		11 (1–30) ^b^		O	No
Fouks et al. (2020) [89]	Total	HC (n = 21) OC (n = 72)	93	15.5 ± 8.3 ^c^	64/93 (68.8%)	879	11 (6–22) ^d^	31.06%				O	No
	Pre-pubertal		33			216	6 (0–16) ^d^	21.3%		2.8 (2.3) ^d^		O	
	Post-pubertal		60			663	10 (5–20.5) ^d^	34.2%		5.6 (4.6) ^d^		O	
Segers et al. (2020) [44]	Total	BC (n = 26) HC (n = 25) OC (n = 21) OD (n = 5)	77	24.5 ± 8.5 ^c^ (0–38) ^b^	76	1220		40.4%		6.7 ± 6.3 ^c^		O + E	Yes
	Pre-pubertal		9					22%					
	Post-pubertal		68					42%					
Karavani et al. (2019) [90]	Total	HC (n = 24) OC (n = 60)	84	(0–18) ^e^	67/84 (79.7%)	736		24.3%				O	No
	Pre-pubertal	HC (n = 5) OC (n = 28)	33	5.4 (4.69) (0–10) ^d,e^	22/33 (66.7%)	226	8.07 ± 6.83 ^c^	15.5%				O	
	Post-pubertal	HC (n = 19) OC (n = 32)	51	16.1 (3.0) (11–18) ^d,e^	45/51 (88.2%)	510	10.77 ± 8.50 ^c^	28.2%				O	
Fasano et al. (2017) [50]	Total	BC (n = 62) HC (n = 37) OC (n = 29) OD (n = 8)	136		105/136 (77.2%)	705		25.9%		139 mature oocytes for 72		O + E	Yes
	Pre-pubertal	HC (n = 5) OC (n = 1)	6	8.7 ± 2.6 ^c^	6/6 (100%)	107	11.5 ± 8.0 ^c^	7.5%				O	
	Post-pubertal	BC (n = 62) HC (n = 32) OC (n = 28) OD (n = 8)	130	27.6 ± 5.6 ^c^	99/130 (76.2%)	598	3.8 ± 4.2 ^c^	23.4%				O	
Abir et al. (2016) [91]	Total	HC (n = 22) OC (n = 19) OD (n = 2)	42	(2–18) ^e^	33/42 (78.2%)	395		30.6%				O	No
	Before treatment	HC (n = 9) OC (n = 11) OD (n = 2)	22	12 ± 5 ^c^ (2–18) ^e^	20/22 (90.9%)	257	12 ± 1 ^c^	32%		4 ± 5		O	
	After treatment	HC (n = 13) OC (n = 7)	20	13 ± 5 ^c^ (2–18) ^e^	13/20 (65%)	138	7 ± 10 ^c^	26.4%		2 ± 3		O	
Yin et al. (2016) [92]	_	BC (n = 20) HC (n = 7) OC (n = 7) OD (n = 2)	36	26 (8–41) ^c^	36 (100%)	393	10.9 ± 9.4 ^c^ (0–43), ^e^	25.1%		NA		O	No
Park et al. (2016) [93]	_	OC (n = 6)	6	29 (19–39) ^f^	5/6	53	10.6 (0–19) ^f^	58.9%		1 to 15		O + E	No
Hourvitz et al. (2015) [77]		BC (n = 4) HC (n = 23) OC (n = 17) OD (n = 12)	56	22.3 ± 1.26 ^a^	30/56 (53%)		6.95 ± 0.83 ^a^	34.7%		2.47 ± 0.41^a^		O + E	No
Takae et al. (2015) [94]	_	BC	27	33.7 ± 3.8 ^c^	25/27 (92.6%)	226	8.3 ± 6.1 ^c^	36.7%		foll phase 3.9 ± 2.8 ^c^, lut phase 3.2 ± 3.7 ^c^		O	No
Escriba et al. (2012) [95]		BC (n = 28) HC (n = 5)	33	31.5 ± 5.2 ^c^	30/33 (90.9%)	108	3.3 ± 0.7 ^c^	36.1%		1.3 ± 0.2 (21/33 patients) ^c^		O	No
Fasano et al. (2011) [96]		BC (n = 26) HC (n = 20) OC (n = 2) OD (n = 9)	57	26 (8–35)	42/57 (73.7%)	266	4	31%				O + E	No
Revel et al. (2009) [97]		HC (n = 8) OC (n = 9) OD (n = 2)	19	15 (5–20) ^e^	17/19 (89.5%)	179	9 (0–37) ^e^	25.1%				O	No
Huang et al. (2008) [35]		BC (n = 2) HC (n = 1) OC (n = 1)	4	21, 35, 18, 38	4 (100%)	11	3, 1, 4, 3	79%		8 total (3, 1, 2, 2)		O	No
Revel et al. (2003) [41]		HC (n = 4) OC (n = 3) OD (n = 2)	9	22.3 ± 6^c^	7/9 (79%)	32	3–7			4.2		O + E	No

^a^: mean ± SEM, ^b^: mean (IQR), ^c^: mean ± SD, ^d^: median (IQR), ^e^: median (min-max), ^f^: total number, BC: breast cancer, HC: hematological cancer, OC: other cancer, OD: other disease, O: oocyte, E: embryo.

## 5. Who Are the Candidates for IVM for Fertility Preservation?

IVM has mainly reported as a method for preserving fertility in cancer patients. However, other indications have also recently emerged [37].

Among cancer patients seeking fertility preservation, breast cancer patients constitute the largest population [98]. Most of the data on IVM for fertility preservation comes from young women diagnosed with breast cancer, mainly involving OPU-IVM (Table 1) in addition to OTO-IVM (Table 2). They face a heightened risk of cancer therapy-induced ovarian insufficiency, compounded by the overall increased incidence of breast cancer in young women [14,99]. Additionally, breast cancer patients exhibit the lowest prospective probability of achieving pregnancy post-cancer treatment for several reasons [15]. These include concerns about pregnancy after breast cancer, recommendations for long-term adjuvant hormonal therapy in hormone receptor-positive patients, and an increased likelihood of hereditary predisposition to cancer among this group. Although it is well-established that ovarian stimulation can be safely performed before adjuvant chemotherapy [100,101], data are more scarce in the neoadjuvant setting. Therefore, most of the evidence on IVM in breast cancer comes from women requiring urgent and/or neoadjuvant chemotherapy. The recent findings in 321 patients indicate that breast cancer characteristics may influence the number of oocytes recovered and the IVM rates [102].

Patients carrying deleterious mutations in breast cancer predisposing genes might request a pre-implantation genetic testing of embryos, which could affect pregnancy chances if no transferrable embryos are obtained. Furthermore, a significant portion of breast cancer patients require urgent neoadjuvant chemotherapy before breast surgery, significantly limiting the window for fertility preservation procedures involving ovarian stimulation, oocyte collection, and vitrification. Consequently, there is a strong advocacy for the development of IVM as an emergency cryopreservation tool in this specific patient population.

Hematologic malignancies in the pediatric and young adult populations often require treatments at risk of alteration of the fertility potential in survivors. Therapies are often urgent, without the possibility of considering ovarian stimulation for oocyte/embryo vitrification. Therefore, ovarian tissue cryopreservation is often presented as the method of choice for young women seeking fertility preservation in the context of hematologic malignancies. Nevertheless, ovarian tissue recovered in leukemia might contain malignant cells, which could be reintroduced at the time of transplantation [103]. Therefore, hematologic diseases such as severe Hodgkin or non-Hodgkin lymphomas and some leukemias may constitutes a realistic indication for IVM [40].

However, the possibility of combining IVM and OTC has recently changed the possible indications of IVM. Not only can it be considered in situations with a high risk of malignant invasion, such as borderline ovarian carcinoma [104], leukemia, neuroblastoma, and Burkitt lymphoma, but also in all candidates for OTC since it may increase the overall chances of success as a result of the additional source of frozen biologic material. In addition, in pre-pubertal girls, for which ovarian tissue cryopreservation has always been considered the sole fertility preservation method, the possibility of recovering oocyte–cumulus complexes from ovarian tissue ex vivo has resulted in a new perspective. However, although theoretically applicable to pre-pubertal children, the full potential of this combined approach remains uncertain, in particular due to limited data on the developmental capacity of immature oocytes from these ovaries.

Some benign diseases requiring possible fertility preservation may also fall on the scope of IVM either due to the need of an urgent treatment and/or the estrogen-dependence of the pathology [44,68,86,91,97], or even as a result of the incapacity of ovaries to respond to exogenous FSH. The latter case is mainly represented by women diagnosed with early-stage autoimmune premature ovarian insufficiency, characterized by the presence of small antral follicles irresponsive to ovarian stimulation. Successful livebirths have been reported in this situation after IVM [66]. Finally, OTO-IVM has also been reported in young Turner syndrome patients [91,97,104,105].

Transmen are more and more often referred for fertility preservation as a result of the potential irreversible impact on the fertility of gender-affirming hormonal treatments. Indeed, the long-term effects of prolonged androgen treatment on future fertility remain poorly understood. Fertility preservation in post-pubertal transmen usually relies on oocyte vitrification after controlled ovarian stimulation. However, some of these men show reluctance and anxiety about the controlled ovarian stimulation-induced hyperestrogenemia, which is likely to exacerbate gender dysphoria. Therefore, Sterling et al. have proposed the use of IVM for fertility preservation in this context [106]. Hence, an immature oocyte yield can be performed transvaginally or ex vivo at the time of ovarian tissue cryopreservation. Data are conflicting regarding the impact of prolonged testosterone treatment on the capacity of immature oocytes to reach the metaphase 2 stage in vitro and its future outcomes. Cumulus–oocyte complexes collected during oophorectomy in 16 transmen exhibited a normal spindle structure and chromosomal alignment. Oocytes that reached metaphase 2 stage did not appear to be morphologically affected by prolonged testosterone treatment [107]. Another investigation reported an oocyte maturation rate of 34.3% and more than 85% of these showed normal spindle structure [108]. However, a recent study in 83 transgender men having taken long-term androgen therapy reported a low developmental capacity of oocytes after IVM, with poor maturation (23.8%) and fertilization rates (34.5%), and frequent early embryo arrest [109]. Importantly, no successful embryo implantation following IVM has been reported in a transman, preventing the ability to draw any conclusions on the efficacy of such procedures in this context.

Moreover, IVM has been reported in different other clinical situations such as during caesarian section in a pregnant woman seeking embryo cryopreservation for future surrogacy in the context of renal transplantation [34] or OTO-IVM during a surgery for ovarian cysts [110].

In a retrospective case–control study, Mamsen et al., reported the results of OTO-IVM in an 18-year-old woman presenting with mosaic Turner syndrome, allowing the cryopreservation of 12 mature oocytes [105].

## 6. How to Improve IVM Success Rate?

One of the current limitations of the transvaginal oocyte aspiration technique is the relatively low and unpredictable recovery rate of oocyte–cumulus complexes from small antral follicles, standing at approximately 50% [111]. Consequently, in tandem with the limited maturation rate of oocytes with current IVM systems, the final number of mature oocytes actually available for fertility preservation is constrained, especially in women with a reduced number of antral follicles or low circulating levels of Anti-Müllerian Hormone (AMH). Sonigo et al. reported that cancer patients with an antral follicle count and AMH values above 28 follicles and 3.9 ng/mL, 20 follicles and 3.7 ng/mL, and 19 follicles and 3.5 ng/mL are required to obtain at least 15, 10, or 8 cryopreserved oocytes, respectively, after standard IVM [78].

In addition, IVM success rate is strongly correlated with the number of immature oocytes recovered [112]. Most of evidence in PCOS patients failed to show any benefit of FSH or hCG priming before immature oocyte aspiration [113]. Recent studies in the context of fertility preservation did not report any improvement in IVM outcomes after hCG or GnRH agonist priming in comparison with unprimed women [70,73]. Based on the lack of available strategies to improve each individual pick-up of oocyte–cumulus complexes, the concept of double-IVM, implying the repetition of IVM cycles even within a very short time frame (<10 days), has emerged as a viable and safe option for increasing the number of mature oocytes available for fertility preservation [64].

Moreover, although a correlation between AMH levels and the ex vivo retrieval of oocytes from extracorporeal ovarian tissue is purported, robust data confirming this correlation are currently lacking. But the combination of IVM and OTC may represent a means of obtaining more gametes to improve fertility preservation results. Theoretically, almost all patients undergoing ovarian biopsies or ovariectomy, except those undergoing chemotherapy before ovarian tissue harvesting, are potential candidates for the combined approach involving IVM of ex vivo retrieved oocytes. Candidates for this combined approach also include patients with borderline ovarian carcinoma or a high risk of malignant ovarian invasion [104].

The lower oocyte competence following IVM when compared to those having undergone in vivo maturation constitutes the main limitation to the implementation of this technique in many labs. The successful maturation of oocytes in vitro depends on both nuclear and cytoplasmic aspects. Beyond nuclear maturation through meiosis I and II, cytoplasmic maturation encompasses crucial steps such as protein and mRNA synthesis, organelle relocation, and the regulation of biochemical processes vital for fertilization and subsequent embryonic development [114]. All the crucial steps leading to optimal nuclear and cytoplasmic maturation are highly modified by the IVM technique. Indeed, oocyte maturation in vitro happens spontaneously upon removal from the follicular environment. The direct consequence is precocious meiotic resumption in immature oocytes. To address this challenge in IVM systems, some suggest delaying spontaneous nuclear maturation while promoting cytoplasmic development simultaneously [114].

**Figure 2:**
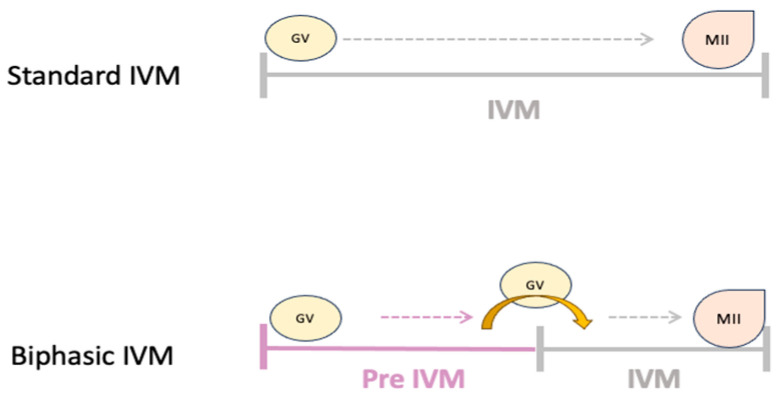
Comparison of standard IVM and biphasic IVM system.

The biphasic IVM system involves two distinct stages: a pre-IVM phase that prevents spontaneous meiotic resumption in oocytes, maintaining communication between oocyte and cumulus cells while enhancing oocyte developmental competence (Figure 2). This is followed by the IVM phase, inducing meiotic resumption and maturation. Hence, cyclic adenosine monophosphate (cAMP) modulators, cyclic guanosine monophosphate modulators, 3-isobutyl-1-methyl-xanthine (IBMX), or c-type natriuretic peptide (CNP) have been used to prevent meiotic resumption. A pre-IVM procedure based on CNP (named “capacitation-IVM” (CAPA-IVM)) has undergone pre-clinical human safety and efficacy trials and its adoption into clinical practice resulted in healthy live birth rates not different from conventional IVF [115,116,117,118]. However, biphasic IVM is still considered experimental. Data on biphasic IVM in the context of ex vivo VM are scarce and have so far been derived from 10 patients who underwent unilateral oophorectomy in the context of gynecological tumors 110]. Ovaries were divided in two equal halves and the COCs from these hemi-ovaries were subjected to standard IVM or CAPAIVM. The biphasic IVM system showed a significantly higher MII rate than standard IVM (56% vs. 35%, respectively) and the only embryos that developed to blastocysts came from CAPAIVM [119].

## 7. Safety in IVM

Concerns arose regarding potential epigenetic reprogramming interference in IVM oocytes [120]. However, normal DNA methylation levels were observed in human oocytes post-IVM [121]. Studies hinting at abnormal methylation in “rescued” immature oocytes from failed conventional IVF cycles do not reflect the IVM context [121]. Furthermore, the occurrences of aneuploidy and chromosomal abnormalities in human embryos from IVM were comparable to standard IVF, affirming IVM’s safety [122].

IVM proves advantageous for women with PCOS by eliminating the risk of ovarian hyperstimulation syndrome [123]. Initially, higher miscarriage rates were noted in IVM, but with a freeze-all approach, the rates became comparable to conventional stimulation [123]. Yet, Belgian studies found increased hypertensive disorder incidences in IVM pregnancies, possibly linked to patients’ severe PCOS phenotype, while preterm birth rates and birth weights remained similar [124].

Notably, singletons from IVM displayed comparable birthweights and preterm birth rates to those from controlled ovarian stimulation [125]. No increased risk of congenital malformations was observed in neonates conceived through IVM. Long-term follow-up studies evaluating potential metabolic disorders and early childhood development in IVM offspring are essential, although current data suggest their safety is akin to standard IVF [126,127,128].

While early childhood studies on IVM offspring show promising outcomes, longer-term investigations are anticipated. The impact of potential epigenetic alterations and heritable changes in ART and, specifically, IVM children remains a critical focus for ongoing research. Importantly, for fertility preservation, follow-up programs monitoring children born from women who underwent IVM are warranted.

## 8. Gaps for Future IVM Research

1. Improving the recovery rate of oocytes in an IVM OPU: Livebirth and cumulative livebirth rates in IVF are strongly correlated to the number of metaphase 2 oocytes. The output rate of immature oocyte recovered is unpredictable and may vary widely from a patient to another, despite similar markers of the ovarian status. The efficiency of IVM would benefit from a higher proportion of cumulus–oocyte complexes yielded. This will require development of novel and sophisticated needle technology.
2. Improving oocyte IVM rate: The development of specific culture systems capable of supporting the growth and differentiation of oocytes prior to meiotic maturation probably represents one of the main issue for the implementation of IVM as a routine practice.
3. Improving embryo development following IVM of oocytes: Animal and human research is needed to improve the in vitro development of embryos derived from oocyte IVM.
4. IVM should be considered as a routine technique for female fertility preservation: Physicians should be aware that IVM alone, OPU-IVM, and OTO-IVM should be integrated in the panel of techniques of fertility preservation techniques. This combination of different methods will be of great benefit for patients.
5. IVM and elective fertility preservation: Due to the convenient, lower cost, and limited stimulation nature of IVM, it provides an alternative for non-medical oocyte vitrification, especially for women with high values of markers of the follicular ovarian status.

## 9. Conclusions

Over the past decade, advancements in oncofertility have reignited interest in IVM due to specific clinical scenarios limiting the feasibility of controlled ovarian stimulation. Immature cumulus–oocyte complexes can be retrieved from small antral follicles at any menstrual cycle phase within a short timeframe. This offers the opportunity to vitrify mature oocytes or embryos post-IVM in urgent situations or when stimulation are not advisable. Extraction methods include transvaginal retrieval or direct laboratory retrieval from extracorporeal ovarian tissue. Despite IVM’s shift from experimental status due to safety studies, it hinges on the intricacies of oocyte maturation. The relatively lower developmental competence of in vitro matured oocytes underscores the imperative to enhance IVM culture systems. Biphasic IVM systems show promise in narrowing the gap in oocyte competence following IVM or ovarian stimulation. Nevertheless, for cancer survivors aiming to optimize conception chances, the preferred option involves combining IVM with ovarian tissue cryopreservation, especially when ovarian stimulation cannot be considered. However, if biphasic IVM confirms its effectiveness, it might constitute a low-intervention, low-cost, safe, and patient-friendly method that is particularly attractive for non-medical fertility preservation.
